# Improving palliative care outcomes for Aboriginal Australians: service providers’ perspectives

**DOI:** 10.1186/1472-684X-12-26

**Published:** 2013-07-23

**Authors:** Shaouli Shahid, Dawn Bessarab, Katherine D van Schaik, Samar M Aoun, Sandra C Thompson

**Affiliations:** 1 Combined Universities Centre for Rural Health (CUCRH), University of Western Australia, M706, 35 Stirling Highway, Crawley, WA, 6009, Australia; 2 Aboriginal Health Education Research Unit, Curtin Health Innovation Research Institute, Curtin University, GPO Box U1987, Perth, WA, 6845, Australia; 3 Daniel C. Tosteson Medical Education Center, Castle Society, Harvard Medical School, 260 Longwood Avenue, Boston, MA, 02115, USA; 4 School of Nursing and Midwifery, Faculty of Health Sciences, Curtin University, GPO Box U1987, Perth, WA, 6845, Australia; 5 Combined Universities Centre for Rural Health, University of Western Australia, 167 Fitzgerald St, PO Box 109, Geraldton, WA, 6531/6530, Australia

**Keywords:** Palliative care, End of life care, Aboriginal, Indigenous, Cultural safety, Australia

## Abstract

**Background:**

Aboriginal Australians have a lower rate of utilisation of palliative care services than the general population. This study aimed to explore care providers’ experiences and concerns in providing palliative care for Aboriginal people, and to identify opportunities for overcoming gaps in understanding between them and their Aboriginal patients and families.

**Methods:**

In-depth, qualitative interviews with urban, rural and remote palliative care providers were undertaken in inpatient and community settings in Western Australia. Interviews were audio-recorded, transcribed verbatim and coded independently by two researchers with QSR NVivo 10 software used to help manage data. Data analysis was informed by multiple theoretical standpoints, including the social ecological model, critical cultural theories and the ‘cultural security’ framework. Thematic analysis was carried out that identified patterns within data.

**Results:**

Fifteen palliative care providers were interviewed. Overall they reported lack of understanding of Aboriginal culture and being uncertain of the needs and priorities of Aboriginal people during end-of-life care. According to several participants, very few Aboriginal people had an understanding of palliative care. Managing issues such as anger, denial, the need for non-medical support due to socioeconomic disadvantage, and dealing with crises and conflicts over funeral arrangements were reported as some of the tensions between Aboriginal patients and families and the service providers.

**Conclusion:**

Early referral to palliative care is important in demonstrating and maintaining a caring therapeutic relationship. Paramount to meeting the needs for Aboriginal patients was access to appropriate information and logistical, psychological and emotional support. These were often seen as essential but additional to standard palliative care services. The broader context of Aboriginal history and historical distrust of mainstream services was seen to impinge on Aboriginal people’s willingness and ability to accept care and support from these services. This context needs to be understood and acknowledged at the system level. More cultural safety training was requested by care providers but it was not seen as replacing the need for an Aboriginal worker in the palliative care team.

## Background

Palliative care considers people’s physical, emotional, psychological and spiritual needs and aims to achieve better quality of life for individual patients, carers and families who are dealing with terminal illnesses and end-of-life processes [1,2]. Lack of understanding of the role of palliative care and the availability of palliative care services, other serious health problems and family issues often rendered palliative care a low-priority issue in Aboriginal^a^ families in which this type of care could have alleviated stressors in caring for a terminally ill member [3]. With the rapid growth of ageing populations and the high burden of chronic and terminal diseases among Aboriginal populations, the need for end-of-life care services is increasing [4]. Comprehensive data on the rates of Aboriginal access to palliative care services are yet to be available in Australia [5]. Data from AIHW (2011) about palliative care separations for Aboriginal and non-Aboriginal Australians indicate that the rate of separation for Aboriginal Australians is higher than non-Aboriginal Australians but that their length of stay is shorter [6]. Although research, mainly conducted in Northern Territory (NT), details the distinctive requirements, values and priorities of Aboriginal people in relation to end of life care and decision-making, there is very little published literature that specifically looks at the provision of palliative care services for Aboriginal people in Western Australia (WA) [7].

A study on palliative care among Aboriginal Australians has identified needs around socio-economic contexts, communication, traditional issues, access to palliative care services, planning and delivery of care, settings of care, workforce issues, information, and training and education [5,8-12]. Services offered by Aboriginal healthcare providers have often had few links with mainstream services. These providers may be unaware of what local palliative care services can offer, resulting in lack of referral of Aboriginal patients to these services. Similarly, mainstream providers may have little awareness either of the special needs of Aboriginal patients or the services, networks and cultural supports available to them. Community members may have little knowledge of palliative care or associate it only with death and dying [13].

Due to their past experiences with the health services and the on-going marginalisation of Aboriginal people within Australian society, many Aboriginal people are fearful toward western health care and use healthcare services reluctantly [5], with many feeling uncomfortable and mistrustful of mainstream health care institutions [8,14,15]. This problem seems more acute when Aboriginal people are facing an incurable illness, as many people prefer to remain in their traditional country or return to their hometown (if a patient is from a rural area) to be cared for by their kin and family members. At this critical stage, returning to their country and family is profoundly important for Aboriginal people as it enables family and community members to visit and say goodbye [9,16]. Thus, it is not surprising that Aboriginal people are underrepresented in the palliative care patient population. Sullivan and colleagues (2003) argued for culturally safe palliative care services, declaring that these services will not be taken up by Aboriginal people unless supported by cultural advocacy and/ or involvement of Aboriginal staff [5].

This research explores key experiences and concerns of palliative care providers (PCPs) in WA when managing treatments and caring for Aboriginal people with a terminal illness. This is predicated on the belief that the perspectives of both Aboriginal patients *and* service providers need to be understood and acknowledged in order to bridge the gaps between them, to improve palliative care access and to ensure development of culturally safe services for Aboriginal people.

## Methods

Ethics approvals and authorization were obtained from Human Research Ethics Committees of Curtin University, WA Country Health Services, tertiary hospitals and the Western Australian Aboriginal Health Ethics Committee.

### Recruitment of participants and collection of data

The views of PCPs were collected as part of a larger study exploring service providers’ perspectives of providing cancer and palliative care treatment and support for Aboriginal patients. The study began in March 2006 and data collection was completed in October 2011. The qualitative method used was exploratory and open-ended. In-depth interviews (face-to-face or by phone) were undertaken with 62 purposively-selected health service providers of cancer and palliative care in Perth and other regional locations in WA. PCPs having experience working with Aboriginal patients were recruited from hospitals, the Cancer Council WA, home-based and local palliative care services, Aboriginal community health and other relevant allied health services. Participants gave voluntary informed consent. This paper reports on 15 interviews with PCPs. The semi-structured interview schedule focused on participants’ experiences with Aboriginal patients in palliative care and their understanding of the barriers and issues experienced by Aboriginal patients and their families.

### Data analysis

The interviews were audio-recorded and transcribed verbatim. Our aim was not to impose any preconceived theories or conceptual framework before data collection. We recorded individual experiences without making prior judgement. QSR NVivo 10 software was used for data management. Analysis began by organising and coding the data into themes which were ordered, recorded and stored as ‘free nodes’ from which other themes were developed through a process of line-by-line reading of the transcriptions [17]. The distribution of important themes were identified, highlighted, labelled, grouped and stored as ‘tree nodes’ which enabled a series of sub-themes to be developed under the key theme. Trustworthiness of the data was ensured through searching for rival explanations and linking the findings and conclusions to data, theory and evidence from the literature [18-20].

During data analysis, we found similarities between the findings from this study and ‘The Living Model’ developed by McGrath and associates in Northern Territory; therefore, the reporting of findings has been partly guided by that model. Seven key principles were identified by McGrath and associates as important for health professionals working with Aboriginal communities in the area of palliative care: equity; autonomy/ empowerment; trust; humane, non-judgemental care; seamless care; emphasis on living; and cultural respect [4,8]. The interpretation and analysis of findings were also guided by the theoretical stance of the social ecological framework [21], the ‘critical cultural approach’ [22] and the ‘cultural security’ framework [23].

## Results and discussion

Among 15 PCPs, 11 were female and four male; seven were from urban areas and eight from rural/remote locations. Three participants were Aboriginal.

An overarching issue for PCPs in WA is that the majority of non-Aboriginal PCPs, regardless of location, mentioned that they do not encounter many Aboriginal patients. One exception was a home and community-based service at a remote location that was run by Aboriginal people and where most of the patients were Aboriginal. Most mainstream PCPs had little experience with Aboriginal patients, and according to them, small numbers affected the opportunities for staff to develop their skills in communication with Aboriginal patients: *“It’s difficult because clearly if we’re not seeing many, then our skill set as a team is not fully developed and optimal”* (Urban, non-Aboriginal). However, while speaking about Aboriginal engagement and service provision, PCPs often failed to recognise the fact that Aboriginal patients would be less likely to access their service unless the service had gained their trust by establishing an ongoing relationship with their community and by addressing cultural issues specific to the community.

The existing literature has also demonstrated low rates of palliative care (PC) utilisation by Aboriginal Australians [24]. The proportion of Aboriginal population living in rural and remote areas in WA and the associated unavailability and inadequate access to specialist PC services in rural/remote WA partially explain this low utilisation [2]. Low utilisation can be the result of past experiences of Aboriginal people within mainstream health services and their vivid memories of previous policies and oppressive treatments from past Australian governments that have created suspicion and mistrust among Aboriginal Australians [8,14]. The importance of trust-building processes has been identified as a core principle for Indigenous Palliative Care Service Delivery Model [8]. Moreover, low PC service utilisation may also be indicative of the need for special cultural needs of Aboriginal Australians which differ significantly from non-Aboriginal cultures and that may not be adequately addressed in mainstream service provision. The establishment of culturally appropriate services may encourage participation and increase accessibility of services that otherwise would not have been utilised by the Aboriginal population [13].

Themes emerging from PCP perspectives were clustered under two key headings: A. Barriers to Aboriginal people utilising palliative care and B. PC providers’ struggles in providing services to Aboriginal people.

### Barriers to Aboriginal people utilising palliative care

Barriers as identified by the PCPs included Aboriginal people’s lack of knowledge and understanding about PC, late referral and a lack of leadership of PC services in the locality.

#### Lack of knowledge and understanding about palliative care

Conceptualizing PC as only related to the ‘last few days of life’ was regarded by the PCPs as the biggest barrier to adequate service utilisation among Aboriginal Australians. Availability of culturally appropriate information and resources may help address these misconceptions. There is evidence that once the concepts, aims and the philosophy of PC were explained to patients, families and health professionals working in rural and remote areas, they positively embraced it [4]. Various methods of communication for reaching Aboriginal communities already exist, such as Indigenous radio stations, National Indigenous Television (NITV), etc. State health departments and PC services, along with other intense community education sessions, need to partner proactively with such media to communicate with and inform Aboriginal communities about PC. Several participants noted that this misunderstanding was also common among non-Aboriginal Australians. As one participant said, PC is often seen as the ‘*last twenty-four hours of life’* when ‘*morphine sedation infusion’* starts. He added, *“I don’t think I’d want to be talking to the palliative care person either if… I saw it was almost like the imminent end of life people”* (Rural, non-Aboriginal). Providers reiterated the importance of a positive portrayal of PC by highlighting how the service can improve the quality of life for people with terminal illness and for their families. As McGrath noted, “a strong palliative care principle of quality of life, or emphasizing the living rather than the dying, is an essential ingredient of end-of-life care” [8]. Such misunderstanding or lack of understanding reflects on the inability of PCPs/ services to promote their services out to the community:

*… our approach is still very much active, care of a person and treatment of a person to manage their pain and to make their end of life experience as comfortable as possible and support for the families. A lot of people still don’t know that or probably appreciate that’s what our service is all about so I would imagine Aboriginal people are no different, perhaps even worse off in terms of understanding what it means to have our service involved and the benefits of it I guess. That to me is the obvious barrier* (Rural, non-Aboriginal).

Several participants expressed doubt as to whether Aboriginal people really wanted them to be there at the critical stages of ‘death and dying’. Non-Aboriginal PCPs sometimes felt that they themselves were pushing Aboriginal people to be in an institutional environment at the end of their lives. One participant denoted PC as an *‘unpleasant necessity’* for Aboriginal people:

*I think that Aboriginal people would see palliative care as, at the very best a really unpleasant necessity at the end of life. … there are bigger and better things to think about. And until you know you’re at that end of life when there’s no other option. I think it’s the last option* (Rural, non-Aboriginal).

This response highlights the dilemma PCPs feel in providing end-of-life care to Aboriginal patients and families. Several of them failed to acknowledge or recognise the impact of the multi-level factors which are highlighted in the socio-ecological model, such as micro level (family, peers, health services), exo (community, media, local politics, social services) and macro (attitudes towards the culture, history, culture) factors, on Aboriginal people’s access to health care services and end up being judgemental about them. The social ecological framework is an overarching interpretive framework that examines and assists in understanding the intertwined relationships among diverse personal and environmental factors to explain a phenomenon [21]. Moreover, Aboriginal perspectives on PC have not been systematically explored in WA and identified as a need. The conclusion that ‘palliative care is an unpleasant necessity’, and that such services are unnecessary for Aboriginal communities, is unproductive and speculative and does nothing to enhance or to promote end of life support for Aboriginal patients.

PCPs acknowledged that care providers often hold a ‘service oriented’ view, using prescribed strategies that are not necessarily appropriate for Aboriginal people and that may result in conflict and dissonance. Moreover, unlike the mainstream western health service culture that treats individuals, Aboriginal culture holds a broad, holistic attitude towards health and wellbeing, in which people want to be treated as a whole person within their sociocultural context.

Participants commented that Aboriginal people from rural and remote locations usually have less understanding of PC. One participant said that PC is equated with Silver Chain (a large domiciliary nursing care service that operates in some states and territories of Australia) and that people *“refuse the Silver Chain service because they think the minute you step into the place [Silver Chain], they’re going to die. And that doesn’t matter whether it’s palliative or not”* (Rural, non-Aboriginal). In a previous study, some Aboriginal patients and families experiencing cancer appreciated the support they received from Silver Chain in relation to administering of medications, and assisting in installation of home-based facilities according to the needs of the patients [25]. This suggests that experiences in different localities and with different Aboriginal families can be quite different.

#### Late referral

Late referral was also seen to affect the access of Aboriginal people to PC services, leading to insufficient time for adequate relationship-building with terminal patients. PCPs thought providers’ lack of understanding of palliative care approaches led to delayed referrals. Linking PC with advanced care planning could help address this issue. PCPs expressed an interest for involvement at an early phase of a person’s disease trajectory. However, they acknowledged that receiving timely referrals was often problematic: *“It may be possible for cancer but for other diseases like renal failure… it can be challenging”* (Rural, non-Aboriginal). This view is supported in the literature, as cancer has a more predictable trajectory than many non-malignant illnesses [26]. According to participants, late referral occurs as a result of geographical dispersion in WA, lack of infrastructure/facilities in rural and remote areas, lack of adequate communication between primary health care providers and PCPs, non-Aboriginal PCPs having restricted access to Aboriginal communities and the unwillingness of Aboriginal people to discuss death and their preferences around dying. PCPs’ comments indicated little understanding that many Aboriginal people do not want non-Aboriginal people coming to their homes for reasons which may include memories of the past when Aboriginal children were removed, judgemental attitude around the lack of quality or well-maintained housing, questions of home ownership, lifestyle and supporting a large extended family, and the lack of peace and privacy at home. PCPs struggle to build relationships with patients referred late, and several providers stated that they needed to concentrate on emergency symptom management rather than holistic patient support. With increased awareness and knowledge of PC in the community, Territory Palliative Care (TPC) have reportedly managed to enhance early referral and improve overall quality of PC in NT [26].

### Palliative care providers’ struggle in delivering services to Aboriginal people

Some participants referred to Aboriginal patients as being gentle and honest, integrating well with the system, easy to work with and grateful and respectful to staff. However, other PCPs found interaction with Aboriginal patients to be particularly stressful and challenging. The following are some of the issues and experiences identified and shared by participants that they felt affected the delivery of end-of-life care to Aboriginal patients and their families: communication; anger around death; family conflict over choices of services; lack of a responsible family carer; obtaining informed consent; cultural differences; and inflexibility of providers with time.

#### Communication

‘Death and dying’ is a sensitive topic for many Aboriginal communities. One participant described a patient who referred to ‘end-of-life discussion’ as *‘bad talk’*. Despite several attempts, the participant failed to continue any conversation with the patient about his needs and preferences at that stage, and the PCP felt frustrated. Without considering the possibilities that patients may not be prepared to face and talk about their own mortality as yet, or may not be ready to engage in such a conversation with a non-Aboriginal stranger, PCPs often come up with a conclusion that ‘*it’s taboo to discuss death in the Aboriginal population*.’ Discussing ‘end-of-life’ issues and practices with Aboriginal patients was particularly difficult for non-Aboriginal providers after a late referral: *“I feel like I’m just really doing more damage than benefit”* (Rural, non-Aboriginal).

Non-Aboriginal service providers often referred to Aboriginal people as being ‘*non-assertive’, ‘non-responsive*’ and unwilling to talk about their preferences, needs, problems and issues: *“I think it’s a very, it’s such a challenging group because there’s so little feedback from the patients themselves about what they understand”* (Rural, non-Aboriginal). Such comments often occurred in the absence of apparent reflection on whether their own communication style facilitated or undermined good communication with Aboriginal people and/or if they were the right person to be having that conversation. PCPs’ preference was to be supported by Aboriginal staff in this critical time, believing that Aboriginal staff are better informed about their own people’s communication style, cultural practices and preferences.

However, not all providers experienced difficulty in conversing about death and dying. Several participants mentioned that they did not have any issue:

*they were very much an Aboriginal family but they operated as anybody else would, there were no issues about talking end of life planning. … when it came to talking to them about end of life planning for him and who’s responsible, I did hesitate a little bit because I wasn’t quite sure how they’d take that, but they were quite fine. They said, oh no, their uncles would deal with all that when the time came, they’d pay for it, they’d organised that they’d step in. So they were very, yes easy to talk to, there were no issues* (Rural, non-Aboriginal).

This quote highlights that there is no set of characteristics identifiable to just one culture or one cultural group but rather diversity among Aboriginal communities and families. Hence, the best approach is to deal with patients as individuals with their individual needs rather than treating them as ‘Aboriginal’ and homogenous. It is also important to note from this conversation that the family had already sorted out the end of life arrangements and so there was no conversation to be had; their response was to inform the service provider that everything was under control and that certain members in the family were already charged with the responsibility of ensuring that certain things happened.

#### Anger around death

Communication was reported as breaking down when some Aboriginal patients and families became angry and resorted to verbal and/or physically aggressive body language. This was seen by PCPs as generally resulting from the pressure Aboriginal people endured in their everyday lives, the frustrations around the diagnosis and prognosis and the associated changes in their lives, and having to leave home to undergo treatments.

*I’m not saying that I’ve ever been physically threatened but… I’ve seen people do things like kick things and yes, so that sort of, or their body language is aggressive. That makes it clearly very difficult to conduct a family meeting when, you know, you’re only the messenger really* (Urban, non-Aboriginal).

Grief and reaction to a poor prognosis was often expressed as non-directed anger. Aboriginal communities routinely experience death and funerals which lead to an accumulation of shock, grief, trauma and sorry time. The high rate of death could be the reason why many Aboriginal people are more likely to be depressed and sometimes angry when they access mainstream services. PCPs reported feeling pressured and struggling to handle these situations effectively. They were conscious that failure to clearly communicate their motives and actions to the patients and their families could increase people’s frustration and anger. In these situations, an Aboriginal Liaison Officer (or health worker), an Elder, a trusted friend or family member can be a crucial support in acting as a go-between the service provider and the patients and their families [8].

#### Family conflict over choices

Negotiating with the family members on issues relating to guardianship, finance, end-of-life care and other socio-cultural concerns were key areas of conflict. Funerals have immense significance within Aboriginal families. Funeral arrangements and discussions on the final resting place of the Aboriginal client were considered as often leading to family arguments at a time when family members were highly stressed and grieving. PCPs expressed the importance of patients writing their last wishes in a will in order to prevent family disputes and to bring a sense of personal autonomy and control at the end of their lives.

*… another thing is a will, they never write wills. None of the patients writes a will. The ones I’ve got anyway. Because sometimes there can be a disagreement of where this person’s going to be buried, either here in [name of the place] or in their home town. But yes, it would make it easier for everyone else if they’d just write it in a will to say where they’re going to be buried, where they want to be buried* (Rural, Aboriginal).

Reportedly, in some families there was conflict between family members when discussing and making decisions about services for sick family members. Support was seen as needed for the grieving families, but these areas of support were quite challenging for non-Aboriginal PCPs.

*Probably the main issue is to support the people who are going to experience most grief, loss and conflict because of death. … the strife that happens after someone dies and property is fought over and the struggles and the conflict that can happen within the family about deciding on a [funeral] service …* (Rural, non-Aboriginal).

This highlights the need for involvement of an interdisciplinary team in patient care that involves a mix of health professionals from a variety of in-patient and community-based organisations. McGrath and colleagues term it ‘seamless care’, comprised of ‘sensitive psycho-social carer’ and ‘skilled clinical carer’ [8]. Aboriginal people often experience lack of information and understanding about the possible consequences of not having a will or are insufficiently informed about where they can go to create a will. Therefore, if service providers can link and refer families to the Public trustee that offers services such as independent, professional trustee provision, asset management, Will and Enduring Power of Attorney drafting, deceased estate administration, executor support, financial administration and trust management services, it may help families to make more informed decisions and minimise the angst and conflict emerging at this time.

#### Family caregiving at end of life

Family members caring for patients at home in some circumstances were labelled as *‘unreliable’* by non-Aboriginal service providers. According to them, this could lead to patients unnecessarily being transferred to hospital at the last stage of their lives:

*the difficulty about the Indigenous patients is finding a carer who’s reliable. And if there is a carer who is reliable they might be able to die at home. But you know…, the best thing about looking after… the Indigenous patients in the country areas, is that they tend to at least die in their country. So we’re mindful of not bringing them back to Perth for unnecessary things* (Rural, non-Aboriginal).

PCPs believed that Aboriginal people would benefit from their services in managing end-of-life care. One participant was quite confident in saying that Aboriginal people die of *‘incurable diseases or terminal illnesses’*, and that they do not stay home for the end-of-life care: “*I think they go into hospitals. I mean that’s just my own quiet opinion. I think they go into hospital because the families won’t do it”* (Urban, non-Aboriginal). Another participant made a similar assumptive comment and referred to having supportive Aboriginal family as ‘*the biggest myth about Aboriginal families’.* He added,

*I don’t know, and maybe the word myth is too strong, but there’s a sense that, you know, the Aboriginal community is tight knit and close [and] they’ll do anything*. *Well I*’*ve seen the opposite to be the case so many times that I no longer believe it to be the case* (Urban, non-Aboriginal).

Such comments require further investigation as they are incongruent with most of the literature and the comments made by other participants in the study. Only a small number of articles have reported Aboriginal people as not willing to care for dying family members; factors identified as reasons included “a lack of commitment to traditional values, fear of ‘blaming’ or ‘payback’, position in the community, concerns about contagion with disease such as cancer, and a reluctance to be involved in the hard work of caring” [NPCP, 2004 in [16]]. This account, though, is not representative of the Aboriginal community as there is a body of literature which supports the notion that Aboriginal people do feel responsible for taking care of their family members while they are sick [10,16,27]. The alternative perception is a good example of how Aboriginal people can be stereotyped or their actions are misinterpreted or misunderstood by mainstream providers and applied to everyone based on the behaviours of a few. In a study with aged and disabled Aboriginal participants, Willis (1999) found almost half of the participants received their principal care from one or more family members and that none of the respondents looked for assistance or support outside their siblings, spouses or descendants in the first and second generations [28]. Many of these families were assisted by community-based health or welfare services. The late or lack of presentation of Aboriginal people to PC services may reflect only situations in which care is beyond that which can be provided in the immediate family, when Aboriginal people need external support services.

Some PCPs appeared to have limited understanding of patients’ logistical challenges, such as difficulty in managing medications and care within the home, absence of local home-based services, local hospitals/ health services lacking enough palliative care beds and other resources, poverty, overcrowded housing conditions and limited facilities in the localities where Aboriginal people live. Some families have other emerging priorities in their lives, such as a mother looking after multiple children or another sick person in her family, on-going grief and loss from family bereavement or not having a quiet place to stay during that critical period of end of life. All of these factors can contribute to Aboriginal patients and their families being unable to take care of a person who is dying, a situation which is quite different to being unwilling, although the end result (i.e., the patient is transferred to a hospital or health service) may be the same. Other research affirms such difficulties being experienced by Aboriginal people and its impact on family care-giving [5,16].

#### Cultural differences

Cultural issues came up as a major barrier for PCPs. Rituals associated with death and dying varied widely, and this variation was considered related to both acculturation and different Aboriginal groups. According to PCPs, taboos and fear of dying made it hard for some family members to see their loved ones die in front of them. PCPs experienced difficulties in arranging and allowing for rituals associated with death, such as collecting and keeping a *‘lock of hair’*, *‘smoking the body and subsequent procedures’*, *‘wailing’*, etc. It was felt that health services should have the provision to address such needs as it was unreasonable and unfair to expect individual PCPs to support patients and families with their multifaceted needs. The need for cultural sensitivity, especially in relation to respect for grief and spiritual practices, access to traditional healers and associated rituals, is well reported in the literature [29,30].

PCPs described Aboriginal people as being very *‘spiritual’* with many reporting that they believe in spirits. Belief in spiritual powers and spirituality impacted patient choice regarding the place and type of care. Service providers discussed payback as a perceived consequence of caring for a dying family member at home:

*a high likelihood of payback for the*…*family members who might have been perceived as being there at the point of death*. *So even*, *a person who*’*s giving care to an Aboriginal person may well be accused of being a contributor to their death*. *So many Aboriginal people and families will want to avoid that level of responsibility and retribution by having the family member in the hospital at the end stage* (Rural, non-Aboriginal).

What is not clear from the above comment is the context in which Aboriginal families may have discussed the issue of payback with them, and this raises the issue of misinterpretation by the non-Aboriginal PCP. While payback is an issue in the community, the context in which people are usually held responsible for the death of someone is when an individual has transgressed and broken a lore which is then seen to be the cause of someone’s death or where they have caused a death through negligence (drunk driving) or a violent act. In areas where traditional cultural beliefs are strong, families will avoid having a family member pass away at home because of their cultural belief system which then requires them to move out of the house sometimes for a very long period of time and sometimes permanently. However, this situation is not constituted as payback and it is important that non-Aboriginal workers understand this. Blaming a family member who is caring for someone who is dying needs to be thoroughly investigated by the service provider, because of the complexity around payback; today the term is often loosely used to explain a situation which in the traditional sense would not constitute payback. While there have been situations when family will attribute a person’s illness to ‘sorcery’, it is usually attributed to someone outside the family and not within. Other reasons for blaming relate to the grief and loss response, in which it is not uncommon for members to blame each other for not taking better care of someone or being responsible for stressing and causing them to worry before their death.

Gender issues can sometimes emerge around palliative care when Aboriginal patients ask to be cared for by a worker of the same sex. If this is not possible, conflict and misunderstanding can occur. PCPs reported that when possible they tried to engage an appropriate person to interact with Aboriginal patients and families:

*actually talking to the patient would be quite different*. *He*’*s an older man and fairly traditional sort of*, *it would be hard for me as a female worker to address those with him*. *And I would probably get the Aboriginal health worker involved* (Rural, non-Aboriginal).

For older Aboriginal men and women, having to be cared for by the opposite sex can cause shame and embarrassment and impact on their dignity, a conflict which highlights and reinforces the need for Aboriginal and non-Aboriginal service providers and key community members to work together to ensure that differing cultural values are respected, sharing is improved and solutions are reached that benefit the patient and ensures that they have quality end of life care. Moreover, as critical cultural theorists point out, most of the health professionals have been oriented to the ‘classic narrow’ view of culture that fails to see the complexity of human experiences within the broader socio-political context and which can lead to a false perception of ‘essentialising’ any culture as “a unified entity… that can be described, taught and understood” [31]. In contrast to this narrow view of culture, appreciation and recognition of culture as a relational entity which changes over time are important, as are identification and differentiation of cultural and non-cultural issues.

#### Informed consent

Participants reported that obtaining ‘informed consent’ was often difficult, especially when patients reached the late stage of their lives. In some instances, participants said the doctors had to make decisions on the patient’s behalf:

*most often the dialysis is planned*, *but sometimes dialysis is commenced as an emergency*, *so how do you get consent in an emergency*? *Do you want to go down to Perth and you*’*ll live or do you want to stay up here and die*? *When a person is sick and nauseated*, *distressed and the family are stressed*, *getting informed consent is next to impossible whether you*’*re white or Aboriginal* (Urban, non-Aboriginal).

Including extended family members in decision making around treatment was challenging and time consuming, particularly when the patient was from a remote community and timely delivery of healthcare to a patient was sometimes compromised. However, service providers need to see the value and to understand the importance of delivering culturally appropriate services to their Aboriginal patients, thereby upholding the core philosophy of palliative care that indicates services need to be provided in the setting preferred by the individual and his/her family and in a culturally acceptable and secure manner [2,32]. Aboriginal patients can feel profoundly distressed when they are treated disrespectfully by health professionals [8].

Health providers need to think about how they can make their services more culturally supportive for Aboriginal people. Engaging with an interdisciplinary approach which includes other service providers could help to reduce the burden of accommodating large families, performance of cultural rituals and practices (33) and knowing whom to contact for assistance with these issues. To ensure culturally appropriate palliative care, the protocols of communal decision-making process need to be respected. Engaging an Aboriginal liaison officer, health care worker within the service or from another service or through video and/ or teleconferencing to assist in facilitating a family decision could also alleviate the difficulties of obtaining consent.

#### Providers not flexible with time

Service providers admitted that they were often too busy to spend additional time with their patients, which made Aboriginal patients and families unhappy. PCPs reported working under tight timeframes, making flexibility difficult. Some participants believed that Aboriginal culture was more relaxed, organic, less time constrained, and less organized. It is to be noted that Aboriginal view of time differs from the Judeo-Christian linear approach, and they have different orientation towards time and priorities [33]. PCPs felt Aboriginal people would prefer informal discussion and for health providers to be flexible and spend extra time with them. As one participant expressed,

… *they* [*Aboriginal patients*] *felt that we weren*’*t flexible enough with time and I think that*’*s because they don*’*t understand that we*’*re not there just to look after that particular person*. *We*’*re there to look after that person at that particular time because after we*’*ve finished here we have to go somewhere else and somewhere else* …. *So that*’*s taken a while for people to learn and that’s always going to be an ongoing issue so we are quite inflexible about times* (Rural, non-Aboriginal).

## Conclusion

This paper has reported the experiences of PCPs in providing palliative care to Aboriginal people, specifically, their opinions and insights on barriers and challenges for Aboriginal people to access palliative care services in WA and their understanding of Aboriginal preferences at end-of-life care and improving services for their Aboriginal patients.

Most service providers shared the view that Aboriginal people, especially at the last stages of their lives, strictly follow cultural practices and rituals. Many non-Aboriginal service providers, although being accepting and understanding of such practices, struggled to accommodate cultural practices within their services. Some PCPs understood the importance of the balance among the physical, emotional, spiritual and mental domains of individual and community life in an Aboriginal context, though this understanding alone was insufficient to facilitate productive relationships with Aboriginal patients in palliative care settings.

Delivering palliative care through the involvement of Aboriginal Community-Controlled Health Services (ACCHS) can also be considered. A recent review article by O’Brien and others (2013) has also suggested this as an approach to open up opportunities for a critical mass of Aboriginal clinicians to be trained and experienced in end of life care and palliation, and to ensure culturally appropriate palliative care service delivery [34]. In many rural and remote areas, the ACCHS are the predominant service providers; however, as they operate on a limited budget, it would not be possible for them to take on other responsibilities without adequate resourcing. ACCHS provide community initiated and led solutions to the deep-rooted social, political and economic conditions that need to be addressed, while contributing to the delivery of essential health care to many Aboriginal communities. They provide a culturally valid model for delivering effective primary health care services to Aboriginal people. (http://www.naccho.org.au/about-us/vision-and-principle/) Aboriginal health professionals interested in palliative care are now being trained under the Program of Experience in Palliative Approach (PEPA) program, thereby increasing opportunities for developing these services in rural and remote WA.

Paramount to addressing the need for access to appropriate information and practical support for Aboriginal patients requiring palliative care is consideration of logistical, psychological and emotional support within palliative care services. The broader socio-cultural context affects Aboriginal people’s willingness and ability to participate in care services and this needs to be understood and acknowledged at the system level. Palliative care embraces a holistic approach to care for both the patient and the family unit, so if practised properly it should resonate well with the Aboriginal culture. More training in cultural security was preferred by care providers, but it was recognised that training does not replace the need for an Aboriginal worker within the palliative care team, or for an Aboriginal-friendly palliative care service.

## Endnote

^a^In this paper, the term Aboriginal has been used to refer to the Indigenous people of Australia. We have used Indigenous when we are referring to features that are identified across different Indigenous peoples around the world.

## Competing interests

The authors declare there are no competing interests, including financial and non-financial.

## Authors’ contributions

SS coordinated the project, participated in the project’s design, carried out the data collection and analysis for this project, and prepared the initial draft. DB, SMA and SCT participated in the design, assisted with the conduct of the study, helped interpret findings, commented upon drafts of the manuscript and writing. KvS was involved in data collection and writing. All authors read and approved the final manuscript.

## Pre-publication history

The pre-publication history for this paper can be accessed here:

http://www.biomedcentral.com/1472-684X/12/26/prepub
